# Supplementary material for: Cryo-EM structure of ex vivo fibrils associated with extreme AA amyloidosis prevalence in a cat shelter
Source: Nat Commun. 2022 Nov 17;13:7041. doi: 10.1038/s41467-022-34743-2 (PMC9672049; doi:10.1038/s41467-022-34743-2)
Supplement: Supplementary file 1 — Supplementary Information [file 41467_2022_34743_MOESM1_ESM.pdf]

SUPPLEMENTARY TABLES AND FIGURES

Supplementary Table 1. Identification of SAA by LC-MS/MS

**Top hits**	
Uniprot entries ranked by relative abundance	(1) Q1T770, Q9XSG7, (2) A0A337SKP2, (3) Q5XXU5
**Data acquisition parameters**	
**Liquid chromatography (LC)**	
UHPLC instrument	Dionex Ultimate 3000
Column	PepMap RSLC C18
(particle, pore sizes, dimensions)	(2 µm, 100 Å, 75 µm x 50 cm)
linear gradients	2-40% formic acid 0.1% in acetonitrile in 70 min and from 40-95% in 10 min,
flow rate	250 nL/min.
**MS/MS**	
Instrument (Company)	Q Exactive (Thermo Fisher Scientific)
Data acquisition	full mass spectra in positive ion mode
m/z range	400-1500
resolution	70000 FWHM
automatic gain control	10^6
maximum injection time	120 ms
Higher-energy C-trap dissociation (HCD)	10 most intense precursor ions
fragment selection (charge states)	(+2, +3 and +4)
normalized collision energy	27%
isolation window	2 m/z
Dynamic exclusion	30 s
Fragment spectra	
resolution	17500 FWHM
automatic gain control	$5*10^4$
maximum injection time	250 ms
**Data processing**	
Software	Proteome Discoverer software, version 2.0
Proteome (Uniprot)	*Felis catus* (UP000011712)
Search settings	Semi-tryptic cleavage
Max missed cleavages	2
Precursor mass tolerance	10 ppm
Fragment mass tolerance	0.03 Da
Static modification	Carbamidomethylation (+57.021) of cysteines
Dynamic modification	M/W Oxidation (+15.995, FDR cut-off < 1)
**PRIDE dataset id**	PXD035851

3 Supplementary Table 2. Cryo-EM data overview

**Data acquisition and processing**	
**Data collection and image processing**	
Microscope (Company)	TALOS Arctica (Thermo Fischer Scientific)
Voltage (kV)	200
Camera	Falcon 3EC
Data collection software	EPU-2.8
Magnification	120,000
Total electron dose ($e^-/Å^2$)	40.0
Defocus range (µm)	-0.8 and -2.2
Pixel size (Å)	0.889
Micrographs (no.)	2,652
**Data processing and reconstruction**	
Software	Relion 3.1 [1,2]
dose-weighting and motion-correction	MOTIONCOR2 [3]
CTF-correction	CTFFIND4 [4]
Helical reconstruction	
Imposed symmetry	C2
Twist / rise	-1.3° / 4.9 Å
Initial segments (no.)	381,233
Segments (no.) in 3D classes with similar cross-sections	335,024
Final segments (no.)	65,122
Phenix auto-sharpening B-factor ($Å^2$)	139.07
**Map/model statistics**	
**Model refinement**	
r.m.s. deviations	
Bond lengths (Å)	0.003
Bond angles (°)	0.941
Ramachandran plot	
Favored (%)	95.95
Allowed (%)	4.05
Outliers (%)	0.00
Validation	
Molprobity score	1.40
Clashscore	3.28
Poor rotamers (%)	0.00
EM-Ringer Score	5.1
Map-model correlation	
CC mask/box/peaks/volume	0.74 / 0.68 / 0.68 / 0.74
**Deposition codes**	
PDB	7ZH7
EMDB	EMD-14726
EMPIAR	EMPIAR-11001

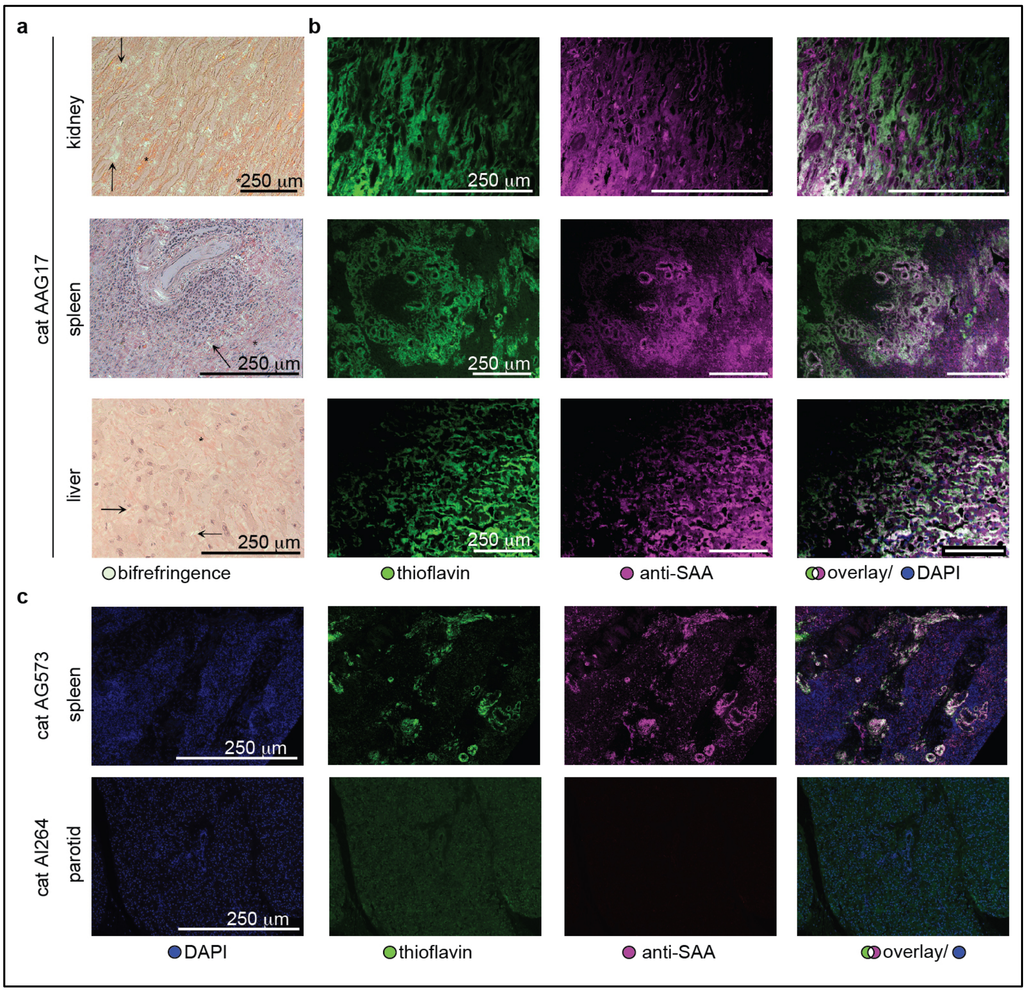

Supplementary Figure 1. AA amyloid in the kidney, spleen and liver of an inflamed cat. (a) After CR staining, amyloid deposits appear orange-red (black asterisks) with green-apple birefringence (black arrows) under polarized light. (b) Thioflavin-S (green) and anti-SAA (magenta) immunofluorescent staining of kidney (top), spleen (middle) and liver (bottom) reveal AA amyloid in all three organs. Co-localized staining at matched relative signal intensity appears white. Nuclei are colored blue and were stained using DAPI. Tissue was obtained from the chronically inflamed cat with ID code AAG17. The cat had reduced red blood cell count (3.8 T/l) as well as increased serum bilirubin (4.6 mg/dL) and creatinine (9.8 mg/dL) concentrations in blood drawn 24h prior death. (c) Spleen and parotid tissue of cats with ID codes AG573 and AI264 were stained positive and negative, respectively, by Thioflavin-S (green) and anti-SAA (magenta). Images were

collected as part of the related study reporting extreme AA amyloidosis prevalence among 80 cats kept in shelters [5].

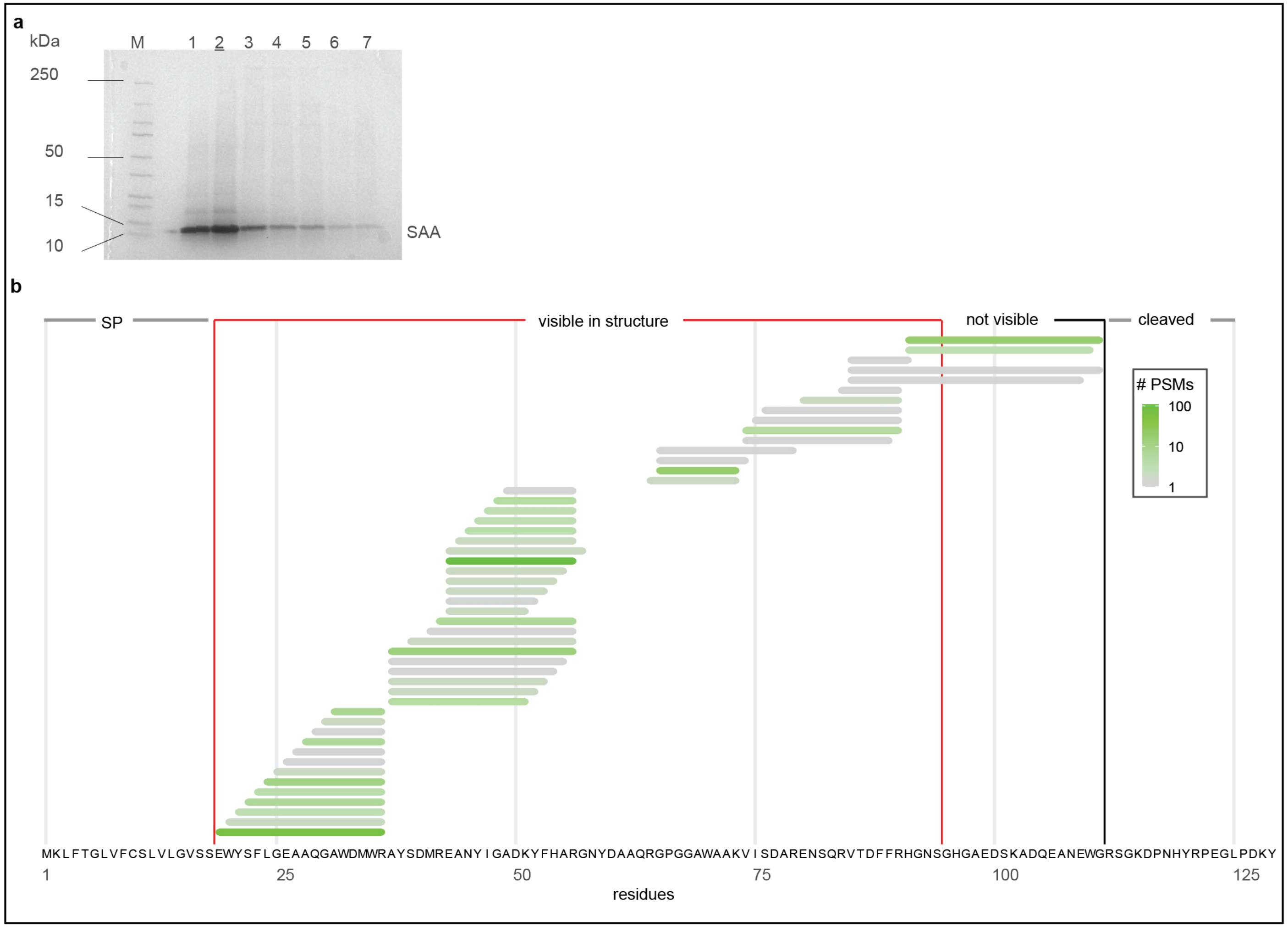

Supplementary Figure 2

Supplementary Figure 2. Most abundant peptides detected by LC-MS/MS match residues 19-111 of the SAA precursor. (a) Coomassie G250-stained denaturing SDS-PAGE revealed the most intense protein bands at a molecular weight between 10 and 15 kDa. Extract run in lane 2 was used for cryo-EM and proteomic analysis. (b) Detected peptides matching Uniprot entry Q1T770 are plotted against the corresponding residue numbers and amino acid single letters. Lines are colored according to the number of peptide spectrum matches (PSM) on a grey to green gradient from 1 to 100 on logarithmic scale. Peptides matching signal peptide (SP) residues 1-18 or C-terminal residues 112-129 were not detected. Residues 19-94, but not 95-111, are visible in the cryo-EM map.

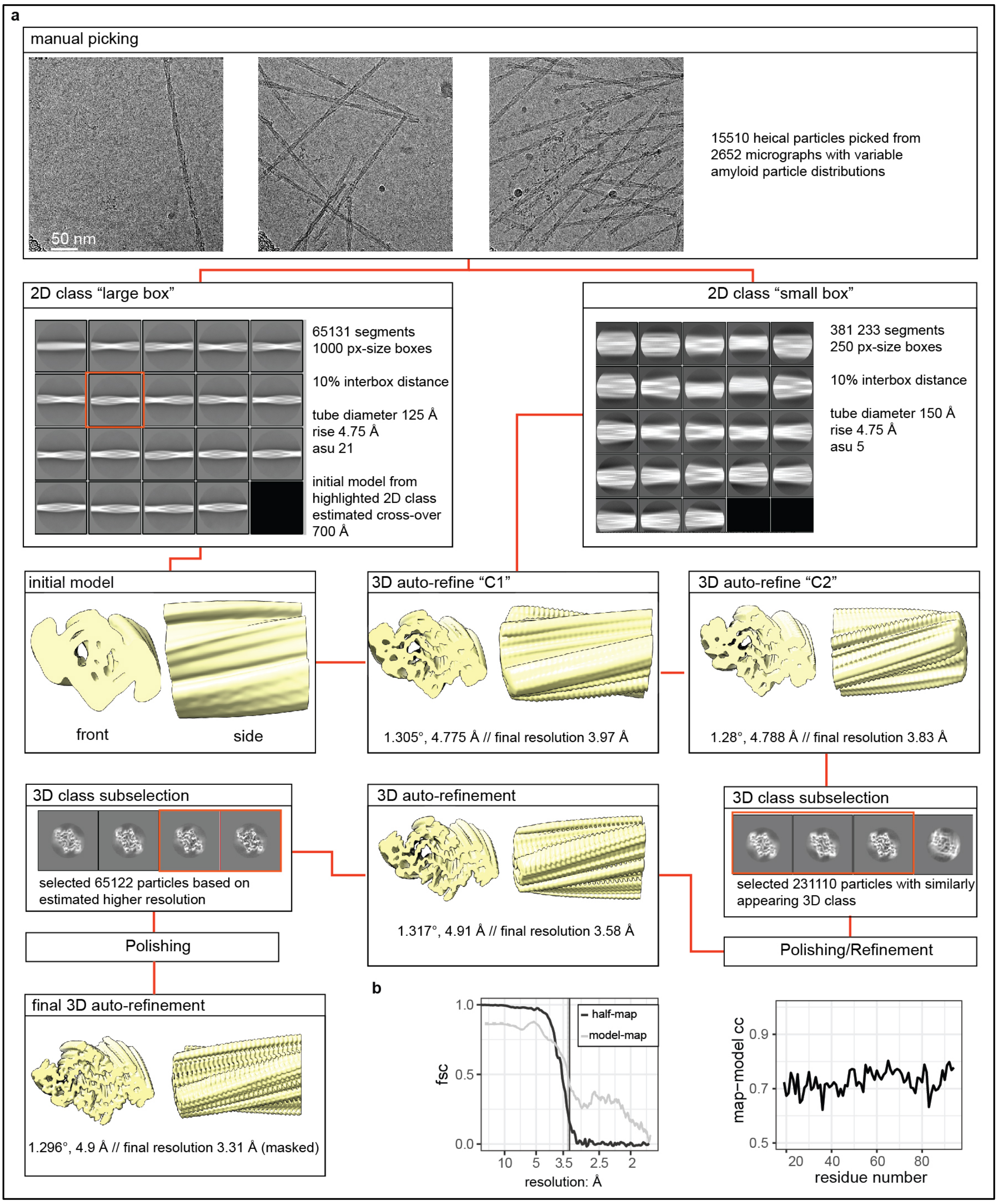

Supplementary Figure 3. Cryo-EM data processing workflow, Fourier shell correlation curves and residue-level map-model cross-correlation plots. (a) The workflow is described in the methods section, following published protocols [2]. Reference-free 2D and 3D class averages are shown as obtained from Relion. For the 2D classes, the number of segments, inter-box distances and helical search parameters (tube diameter, rise, asymmetric unit (asu)) are listed. In 2D and 3D class sub-selection, class averages

highlighted in red were selected for further processing. To visualize the improvement in map quality, yellow colored maps are shown in front and side views, along with listed values for the estimated resolution as well as helical twist and rise parameters. Bayesian Polishing and per-particle CTF refinement steps were added at indicated steps during the workflow. (b) Half-map (black) and model-map (grey) based Fourier shell correlation (FSC) curves from EMDataBank and Phenix [6,7] yield global resolution estimates of 3.3 Å. A residue-level map-model cross-correlation plot reveals map support for all residues, with a mean value of 0.73 ± 0.04. Map-model CC values of >0.7 and <0.5 are thought of as good and poor fits, respectively [7].

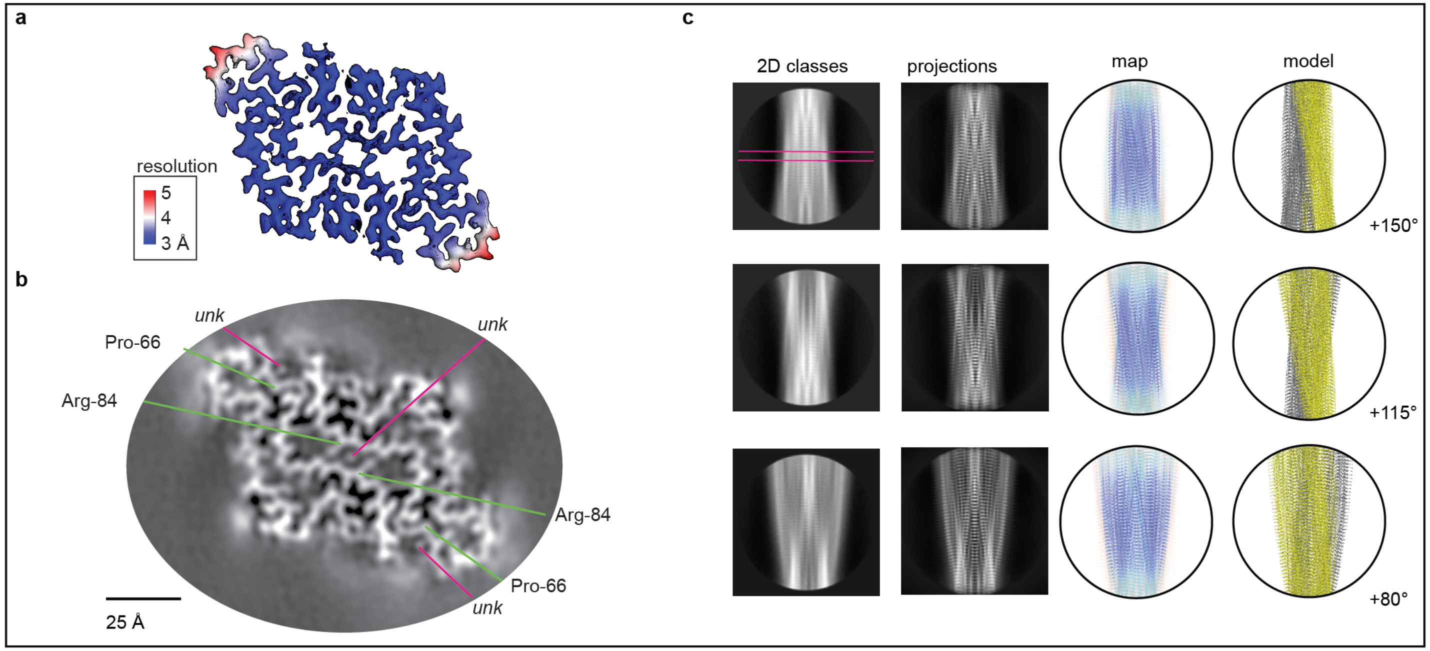

Supplementary Figure 4

Supplementary Figure 4. 2D class averages, indicate a consistent reconstruction yielding a model with definitive map support. (a) The map is colored on a blue, white and red color gradient corresponding to local resolution estimates of 3, 4 and 5 Å, respectively. (b) Cross-section view of the reconstructed map as averaged sum of four XY-slices on grey scale (top, “Relion slices”). Map features outlining unknown (UNK) ligands/ions are highlighted in pink. Residues Pro-66 and Arg-84 are labeled in green. (c) Images of three additional 2D class averages are compared to the 2D projections and volume representations of the reconstructed map, as well as model visualizations as in Figure 2a. Map orientations were matched by applying the known rotation angles from the 2D projections. Rotation angles to turn the fibril around the long axis, relative to the orientation in Figure 2D, are indicated. Thin red lines were added to the top 2D class average to highlight the non-staggered rung levels. Staggering would be expected for pseudo-2_1 symmetry, as described previously [8].

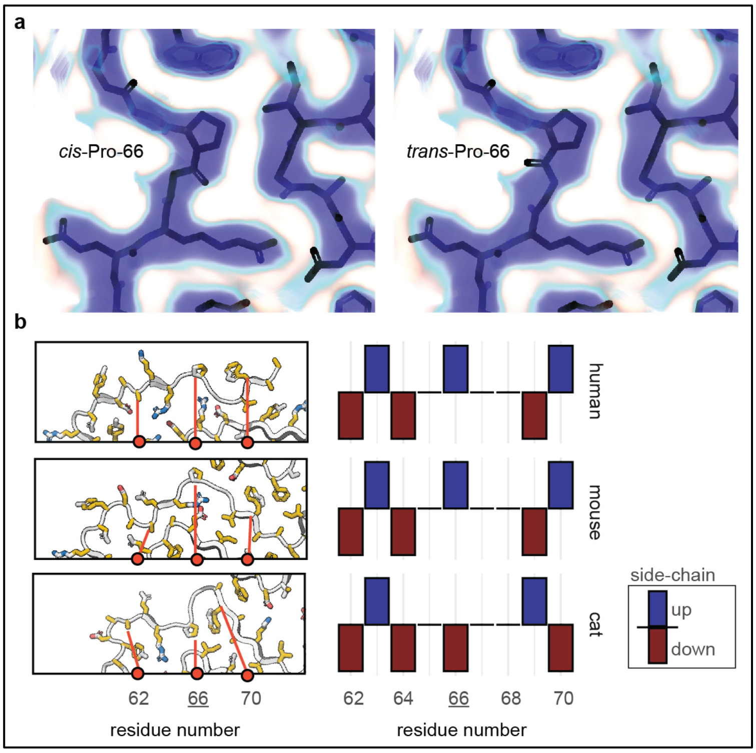

Supplementary Figure 5. *cis*-Proline is supported by map and side chain pointing directions of following residues. (a) As test Pro-66 was modeled both as *trans* and *cis*-isomer with similar Molprobity quality statistics, but the *cis*-isomer appeared as a better fit to the map. Map contours were colored as in Figure 2b. (b) The side chains of residues following Pro66 in cat AA amyloid point to opposite directions compared to mouse and human AA amyloid, where Pro-66 is in *trans* conformation. The side chain pointing directions are shown for human, mouse and cat AA amyloid from top to bottom in two separate panels. (left panel) Residues 62-70 are visually aligned to compare side chain pointing directions. Up- and down directions are defined based on the backbone shown as cartoon. (right panel) Upwards, and downwards pointing side chains are shown in blue and red.

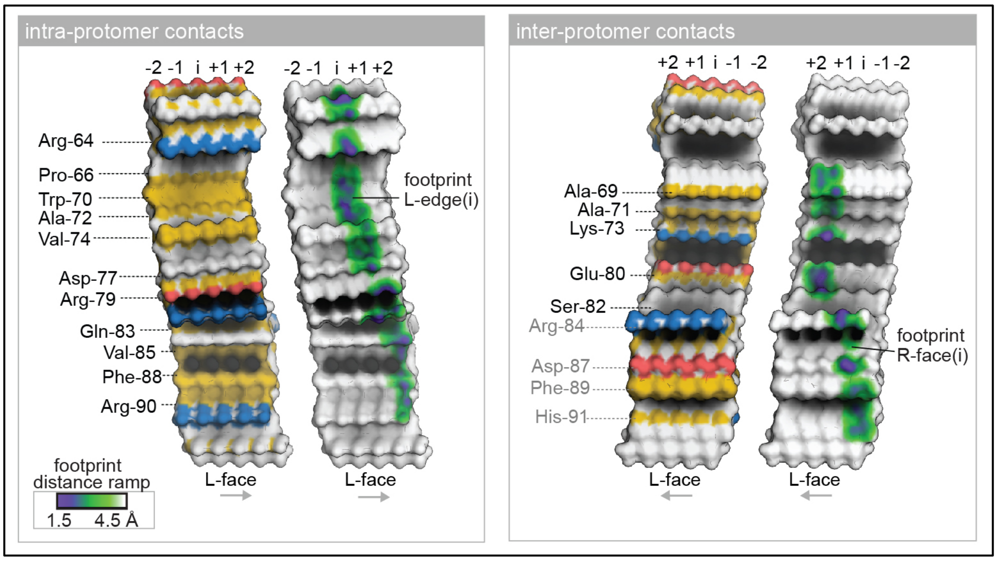

Supplementary Figure 6. Footprints of central rungs on the face sheet at the intra- and inter-protomer interfaces. Molecular footprints illustrate the staggered contacts between the central edge and face rungs at the intra- and inter-protomer interfaces, respectively. The molecular surfaces are colored (left) as in Figure 3, and (right) on a blue-green color-ramp based on the distances to the opposing central rungs.

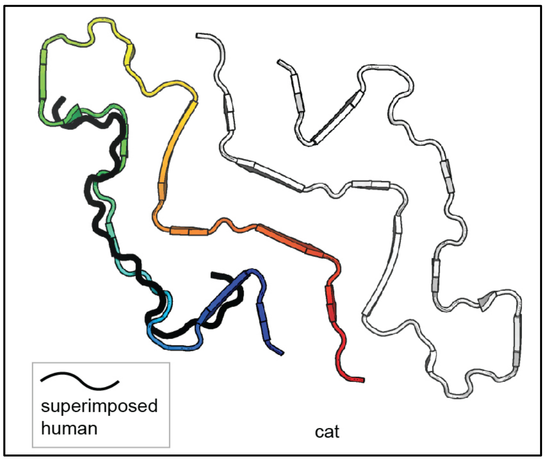

Supplementary Figure 7. Short structural element of human AA amyloid can be superimposed on cat AA amyloid structure. A single 20-residue long segment from residues 24 to 54 of human AA amyloid is superimposed on cat AA amyloid with a rmsd-value of 2.5 Å, and shown as black ribbon.

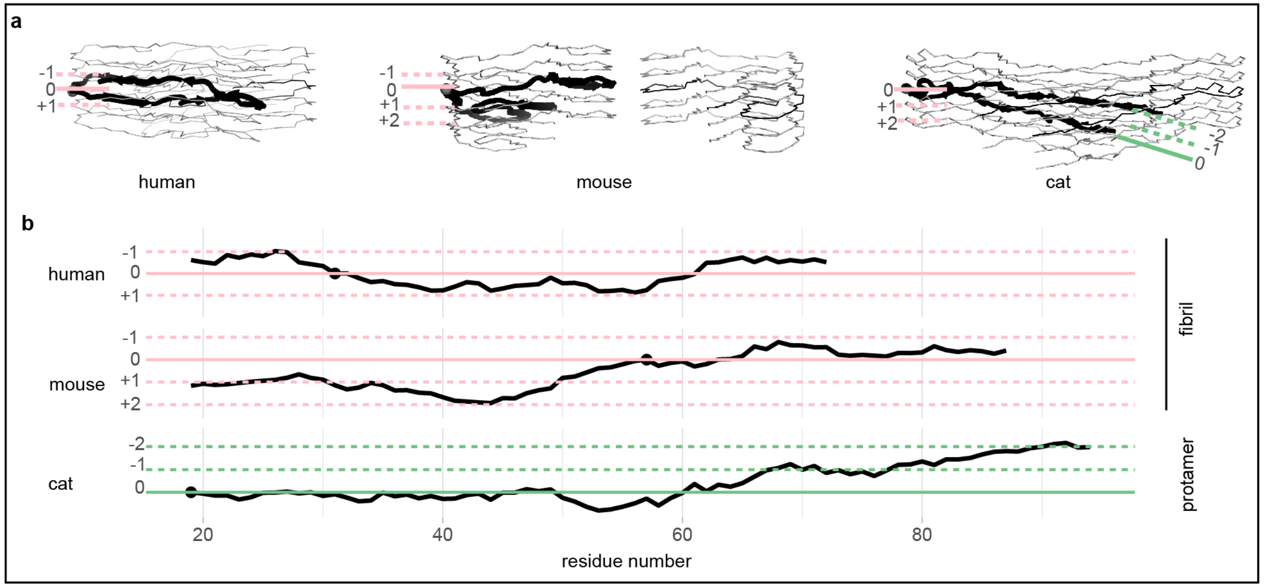

Supplementary Figure 8

Supplementary Figure 8. Layer level crossings of non-planar rungs in human, mouse and cat AA amyloid. The rungs of all three fibrils are non-planar and their backbone Cα-positions cover distance ranges of 11, 13.5 and 16.5 Å along the long axes of the human, mouse and cat fibrils (a) Same view as in Figure 4c, but the chain is shown in black. (b) In case of the human and mouse fibrils, the position of backbone Cα atoms of a single chain is plotted along the long fibril axis (red coordinate system). The layer lines are based on the Cα positions of Ala-31 and Gly-57. Due to the large tilt angles in the cat fibril, the Cα-positions of the left protomer are turned by 14° to re-orient the N-terminal tail perpendicular to the viewing y-axis (green coordinate system).

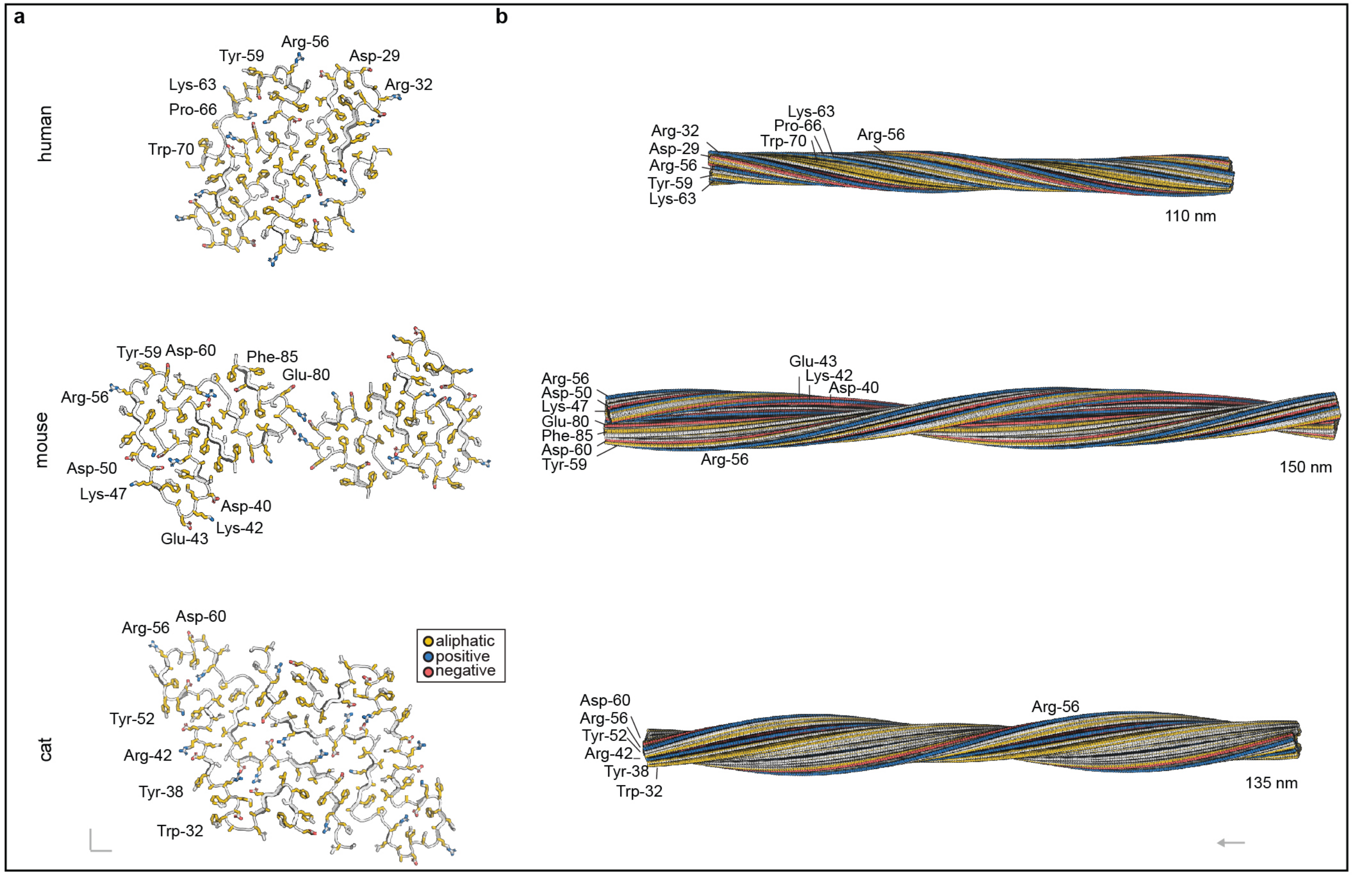

Supplementary Figure 9

Supplementary Figure 9. Distinct intra- and inter-protomer interfaces and fibril architectures of human, mouse and cat AA amyloid. (a) The distinct intra- and inter-protomer interfaces of each fibril are apparent in cross-section views of single rungs in cartoon/stick format on yrb-scale. Residues accessible on the surface of assembled fibrils are labeled. (b) Assembly of the distinct rungs into fibrils yields structures with distinct surface chemical properties. Surface-accessible residues are labeled as in panel a. Pitch lengths are indicated.

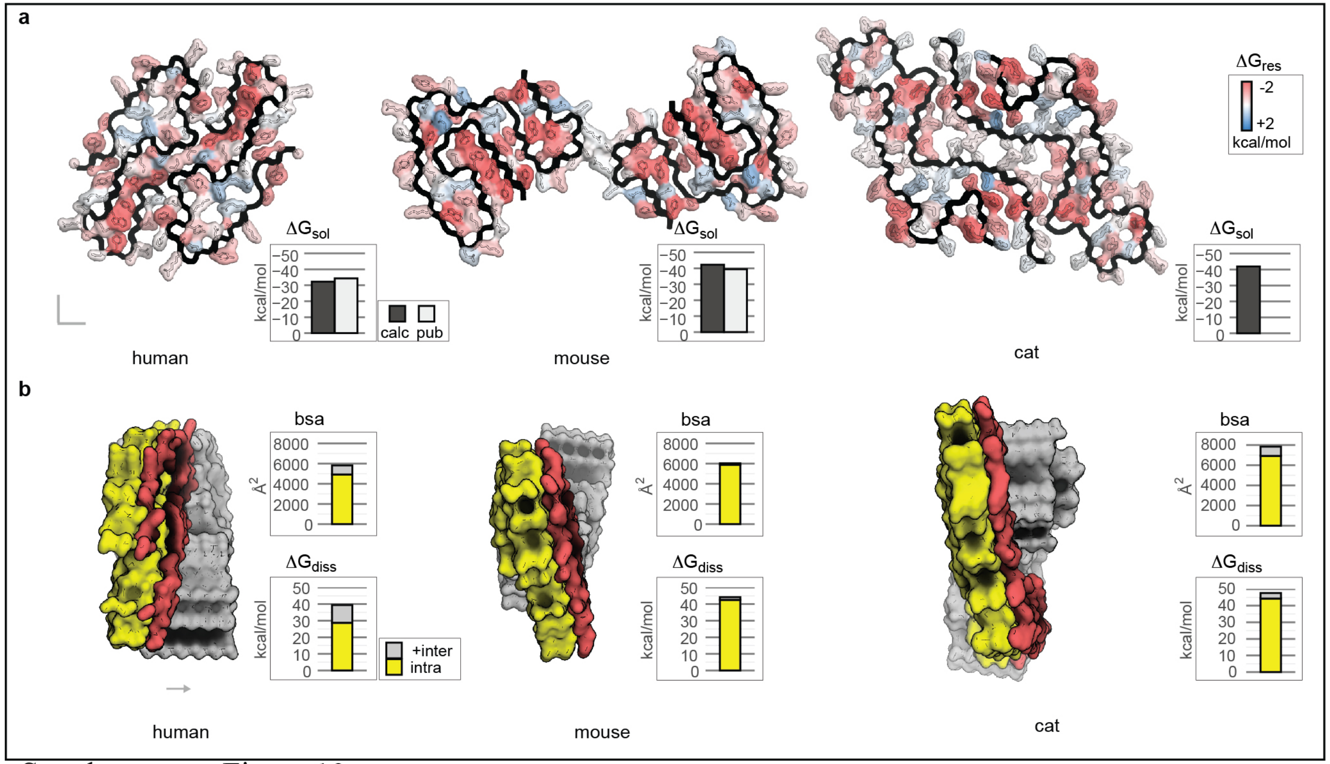

Supplementary Figure 10

Supplementary Figure 10. Eight-residue insert contributes to increased cat AA amyloid fibril core mass, buried surface area and dissociation free energy.

(a) Residue-level solvation free energies (ΔG_{res}) were calculated and visualized following published guidelines [9]. Stabilizing and destabilizing residues are colored on a red-white-blue gradient according to the calculated ΔG residue values between -2 and +2 kcal/mol. Bar-plots reveal that the calculated (dark-grey) molecular solvation free energies (ΔG_{sol}) closely match published values [9] (white) of human and mouse AA amyloid. (b) Side views for the assembly of single chains (red) within each proto-filament (yellow) and fibril (yellow+grey) visualize the intra- and inter-molecular interaction surfaces within human, mouse and cat AA amyloid. Bar plots compare buried surface areas (bsa) and dissociation free energies (ΔG_{diss}) of single chains within each proto-filament (yellow) and the additional contributions of the inter-protomer interface (grey). Values were obtained from PISA[10].

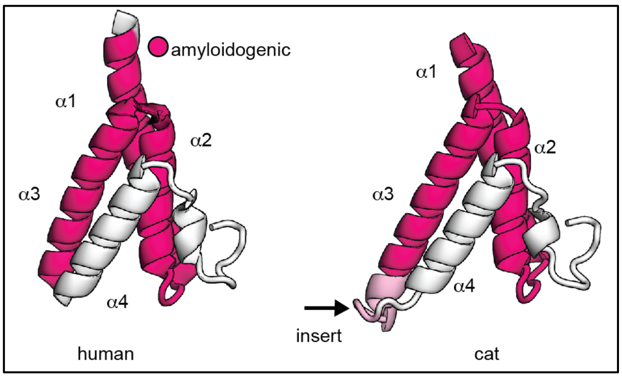

Supplementary Figure 11. Local conformational changes in the predicted structure of native lipid-free cat SAA. (left) Native human SAA adopts a four-helix (α1-α4) bundle structure (PDB: 4IP9) [11]. The amyloid-forming segment is highlighted in magenta. (right) The AI-based model [12] of native cat SAA differs in the conformations of the start and end segments of helices α4 and α3, respectively, as well as of the connecting loop, due to the presence of the eight-residue insert shown in pink.

References

1. Scheres, S. H. W. RELION: Implementation of a Bayesian approach to cryo-EM structure determination. *J Struct Biol* **180**, 519–530 (2012).
2. Scheres, S. H. W. Amyloid structure determination in RELION-3.1. *Acta Crystallogr. D* **76**, 94–101 (2020).
3. Zheng, S. Q. *et al.* MotionCor2: anisotropic correction of beam-induced motion for improved cryo-electron microscopy. *Nat. Methods* **14**, 331–332 (2017).
4. Rohou, A. & Grigorieff, N. CTFFIND4: Fast and accurate defocus estimation from electron micrographs. *J. Struct. Biol.* **192**, 216–221 (2015).
5. Ferri, F. *et al.* AA-amyloidosis in cats (Felis catus) housed in shelters. *bioRxiv* **2022.05.04.490646**, (2022).
6. Lawson, C. L. *et al.* EMDataBank unified data resource for 3DEM. *Nucleic Acids Res.* **44**, D396-403 (2016).
7. Afonine, P. V. *et al.* New tools for the analysis and validation of cryo-EM maps and atomic models. *Acta Crystallogr. D* **74**, 814–840 (2018).
8. Liberta, F. *et al.* Cryo-EM fibril structures from systemic AA amyloidosis reveal the species complementarity of pathological amyloids. *Nat. Commun.* **10**, 1104 (2019).
9. Sawaya, M. R., Hughes, M. P., Rodriguez, J. A., Riek, R. & Eisenberg, D. S. The expanding amyloid family: Structure, stability, function, and pathogenesis. *Cell* **184**, 4857–4873 (2021).
10. Krissinel, E. Macromolecular complexes in crystals and solutions. *Acta Crystallogr. D* **67**, 376–385 (2011).

11. Lu, J., Yu, Y., Zhu, I., Cheng, Y. & Sun, P. D. Structural mechanism of serum amyloid A-mediated inflammatory amyloidosis. *Proc. Natl. Acad. Sci. U.S.A.* **111**, 5189–5194 (2014).

12. Jumper, J. *et al.* Highly accurate protein structure prediction with AlphaFold. *Nature* **596**, 583–589 (2021).
